# 2-Chloro­benzo[*h*]quinoline-3-carbaldehyde

**DOI:** 10.1107/S1600536809040720

**Published:** 2009-10-10

**Authors:** S. Mohana Roopan, F. Nawaz Khan, R. Subashini, Venkatesha R. Hathwar, Seik Weng Ng

**Affiliations:** a Chemistry Division, School of Science and Humanities, VIT University, Vellore 632 014, Tamil Nadu, India; b Solid State and Structural Chemistry Unit, Indian Institute of Science, Bangalore 560 012, Karnataka, India; c Department of Chemistry, University of Malaya, 50603 Kuala Lumpur, Malaysia

## Abstract

The benzo[*h*]quinolinyl fused-ring of the title compound, C_14_H_8_ClNO, is planar (r.m.s. deviation = 0.016 Å); the formyl group is slightly bent out of the plane [the C—C—C—O torsion angle is 10.7 (4)°].

## Related literature

For a review of the synthesis of quinolines by the Vilsmeier–Haack reaction, see: Meth-Cohn (1993 ▶).
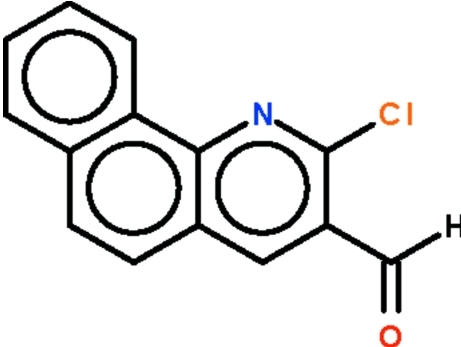


## Experimental

### 

#### Crystal data

C_14_H_8_ClNO
*M* *_r_* = 241.66
Monoclinic, 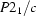

*a* = 3.9833 (2) Å
*b* = 12.4722 (6) Å
*c* = 21.4561 (13) Å
β = 90.687 (6)°
*V* = 1065.87 (10) Å^3^
*Z* = 4
Mo *K*α radiation
μ = 0.34 mm^−1^
*T* = 290 K
0.20 × 0.15 × 0.15 mm

#### Data collection

Oxford Diffraction Excalibur diffractometer
Absorption correction: multi-scan (*CrysAlis Pro*; Oxford Diffraction, 2009 ▶) *T*_min_ = 0.936, *T*_max_ = 0.951
12099 measured reflections
1872 independent reflections
935 reflections with *I* > 2˘*I*)
*R*_int_ = 0.093

#### Refinement

*R*[*F* ^2^ > 2σ(*F* ^2^)] = 0.041
*wR*(*F* ^2^) = 0.087
*S* = 0.81
1872 reflections
154 parameters
H-atom parameters constrained
Δρ_max_ = 0.14 e Å^−3^
Δρ_min_ = −0.19 e Å^−3^

### 

Data collection: *CrysAlis Pro* (Oxford Diffraction, 2009 ▶); cell refinement: *CrysAlis Pro*; data reduction: *CrysAlis Pro*; program(s) used to solve structure: *SHELXS97* (Sheldrick, 2008 ▶); program(s) used to refine structure: *SHELXL97* (Sheldrick, 2008 ▶); molecular graphics: *X-SEED* (Barbour, 2001 ▶); software used to prepare material for publication: *publCIF* (Westrip, 2009 ▶).

## Supplementary Material

Crystal structure: contains datablocks global, I. DOI: 10.1107/S1600536809040720/tk2551sup1.cif

Structure factors: contains datablocks I. DOI: 10.1107/S1600536809040720/tk2551Isup2.hkl

Additional supplementary materials:  crystallographic information; 3D view; checkCIF report

## Figures and Tables

**Fig. 1.**
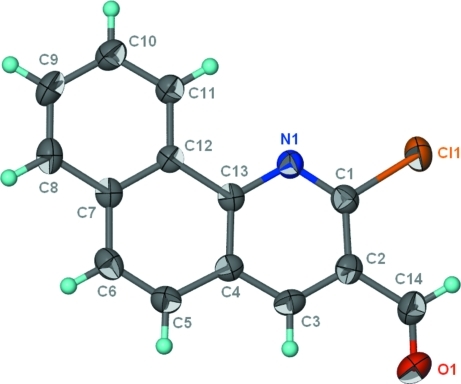
Thermal ellipsoid plot (Barbour, 2001) of C14H8ClNO at the 50% probability level; hydrogen atoms are drawn as spheres of arbitrary radius.

## supplementary crystallographic information

### Experimental

A Vilsmeier-Haack adduct prepared from phosphorus oxytrichloride (6.5 ml, 70 mmol) and *N*,*N*-dimethylformamide (2.3 ml, 30 mmol) at 273 K was added to *N*-(1-naphthyl)acetamide (1.85 g, 10 mmol), and the mixture was heated at 353 K for 15 h. The mixture was then poured onto ice, and the white product was collected and dried. The compound was purified by recrystallization from a petroleum ether/ethyl acetate mixture.

### Refinement

Carbon-bound H-atoms were placed in calculated positions (C–H 0.93 Å) and were included in the refinement in the riding model approximation with *U*_iso_(H) set to 1.2*U*_eq_(C).

### Crystal data

**Table:**

C_14_H_8_ClNO	*F*(000) = 496
*M**_r_* = 241.66	*D*_x_ = 1.506 Mg m^−^^3^
Monoclinic, *P*2_1_/*c*	Mo *K*α radiation, λ = 0.71073 Å
Hall symbol: -P 2ybc	Cell parameters from 1012 reflections
*a* = 3.9833 (2) Å	θ = 1.9–20.4°
*b* = 12.4722 (6) Å	µ = 0.34 mm^−^^1^
*c* = 21.4561 (13) Å	*T* = 290 K
β = 90.687 (6)°	Block, colorless
*V* = 1065.87 (10) Å^3^	0.20 × 0.15 × 0.15 mm
*Z* = 4	

### Data collection

**Table:**

Oxford Diffraction Excalibur diffractometer	1872 independent reflections
Radiation source: fine-focus sealed tube	935 reflections with *I* > 2˘*I*)
graphite	*R*_int_ = 0.093
ω scans	θ_max_ = 25.0°, θ_min_ = 3.3°
Absorption correction: multi-scan (*CrysAlis PRO*; Oxford Diffraction, 2009)	*h* = −4→4
*T*_min_ = 0.936, *T*_max_ = 0.951	*k* = −14→14
12099 measured reflections	*l* = −25→25

### Refinement

**Table:**

Refinement on *F*^2^	Primary atom site location: structure-invariant direct methods
Least-squares matrix: full	Secondary atom site location: difference Fourier map
*R*[*F*^2^ > 2σ(*F*^2^)] = 0.041	Hydrogen site location: inferred from neighbouring sites
*wR*(*F*^2^) = 0.087	H-atom parameters constrained
*S* = 0.81	*w* = 1/[σ^2^(*F*_o_^2^) + (0.0379*P*)^2^] where *P* = (*F*_o_^2^ + 2*F*_c_^2^)/3
1872 reflections	(Δ/σ)_max_ = 0.001
154 parameters	Δρ_max_ = 0.14 e Å^−^^3^
0 restraints	Δρ_min_ = −0.19 e Å^−^^3^

### Special details

**Table:**

Geometry. All e.s.d.'s (except the e.s.d. in the dihedral angle between two l.s. planes) are estimated using the full covariance matrix. The cell e.s.d.'s are taken into account individually in the estimation of e.s.d.'s in distances, angles and torsion angles; correlations between e.s.d.'s in cell parameters are only used when they are defined by crystal symmetry. An approximate (isotropic) treatment of cell e.s.d.'s is used for estimating e.s.d.'s involving l.s. planes.

### Fractional atomic coordinates and isotropic or equivalent isotropic displacement parameters (Å^2^)

**Table:**

	*x*	*y*	*z*	*U*_iso_*/*U*_eq_	
Cl1	0.74845 (19)	0.87817 (5)	0.54210 (3)	0.0598 (3)	
O1	0.1454 (5)	0.63739 (16)	0.45218 (9)	0.0658 (6)	
N1	0.7413 (5)	0.74695 (15)	0.63554 (10)	0.0385 (6)	
C1	0.6418 (6)	0.75706 (19)	0.57774 (13)	0.0390 (7)	
C2	0.4576 (6)	0.6809 (2)	0.54353 (12)	0.0380 (7)	
C3	0.3790 (6)	0.58737 (19)	0.57384 (12)	0.0399 (7)	
H3	0.2557	0.5348	0.5531	0.048*	
C4	0.4818 (6)	0.57031 (19)	0.63539 (12)	0.0347 (7)	
C5	0.4052 (6)	0.4751 (2)	0.66944 (13)	0.0414 (7)	
H5	0.2857	0.4201	0.6501	0.050*	
C6	0.5043 (6)	0.4643 (2)	0.72937 (13)	0.0431 (7)	
H6	0.4504	0.4018	0.7506	0.052*	
C7	0.6893 (6)	0.5458 (2)	0.76127 (12)	0.0360 (7)	
C8	0.7886 (6)	0.5348 (2)	0.82389 (13)	0.0473 (8)	
H8	0.7339	0.4725	0.8453	0.057*	
C9	0.9636 (7)	0.6135 (2)	0.85384 (13)	0.0513 (8)	
H9	1.0257	0.6051	0.8955	0.062*	
C10	1.0497 (6)	0.7069 (2)	0.82201 (13)	0.0474 (7)	
H10	1.1693	0.7606	0.8426	0.057*	
C11	0.9598 (6)	0.7202 (2)	0.76084 (12)	0.0388 (7)	
H11	1.0219	0.7822	0.7399	0.047*	
C12	0.7750 (6)	0.64093 (19)	0.72954 (12)	0.0319 (6)	
C13	0.6639 (6)	0.65335 (19)	0.66560 (12)	0.0335 (7)	
C14	0.3425 (7)	0.6954 (2)	0.47811 (14)	0.0496 (8)	
H14	0.4283	0.7530	0.4558	0.060*	

### Atomic displacement parameters (Å^2^)

**Table:**

	*U*^11^	*U*^22^	*U*^33^	*U*^12^	*U*^13^	*U*^23^
Cl1	0.0849 (6)	0.0483 (5)	0.0463 (5)	−0.0102 (4)	−0.0001 (4)	0.0101 (4)
O1	0.0809 (16)	0.0710 (15)	0.0450 (14)	−0.0128 (12)	−0.0151 (12)	−0.0047 (12)
N1	0.0463 (15)	0.0353 (14)	0.0341 (15)	−0.0004 (10)	0.0016 (11)	−0.0006 (11)
C1	0.0404 (17)	0.0381 (16)	0.0387 (18)	−0.0002 (13)	0.0065 (14)	−0.0006 (14)
C2	0.0378 (17)	0.0421 (17)	0.0342 (18)	0.0034 (14)	0.0058 (14)	−0.0006 (14)
C3	0.0380 (16)	0.0431 (18)	0.0386 (18)	−0.0005 (13)	−0.0012 (14)	−0.0101 (14)
C4	0.0341 (16)	0.0356 (16)	0.0344 (18)	0.0033 (13)	0.0031 (13)	−0.0056 (14)
C5	0.0420 (17)	0.0370 (17)	0.0452 (19)	−0.0014 (13)	−0.0003 (15)	−0.0032 (15)
C6	0.0406 (17)	0.0362 (16)	0.053 (2)	0.0025 (14)	0.0051 (15)	0.0069 (15)
C7	0.0339 (16)	0.0377 (16)	0.0367 (18)	0.0071 (13)	0.0059 (14)	0.0009 (14)
C8	0.0514 (19)	0.0481 (18)	0.043 (2)	0.0077 (15)	0.0037 (15)	0.0110 (15)
C9	0.059 (2)	0.064 (2)	0.0311 (17)	0.0077 (17)	−0.0036 (15)	0.0004 (17)
C10	0.0527 (19)	0.0489 (18)	0.040 (2)	0.0048 (15)	−0.0053 (15)	−0.0048 (16)
C11	0.0417 (17)	0.0360 (16)	0.0385 (18)	0.0054 (13)	−0.0002 (14)	−0.0013 (14)
C12	0.0281 (15)	0.0329 (16)	0.0349 (17)	0.0069 (12)	0.0017 (13)	−0.0020 (13)
C13	0.0314 (16)	0.0347 (16)	0.0347 (17)	0.0045 (12)	0.0057 (13)	−0.0003 (13)
C14	0.058 (2)	0.053 (2)	0.0383 (19)	0.0027 (16)	0.0012 (16)	0.0024 (16)

### Geometric parameters (Å, °)

**Table:**

Cl1—C1	1.748 (3)	C6—H6	0.9300
O1—C14	1.200 (3)	C7—C8	1.403 (3)
N1—C1	1.303 (3)	C7—C12	1.412 (3)
N1—C13	1.371 (3)	C8—C9	1.361 (3)
C1—C2	1.402 (3)	C8—H8	0.9300
C2—C3	1.374 (3)	C9—C10	1.395 (3)
C2—C14	1.483 (3)	C9—H9	0.9300
C3—C4	1.394 (3)	C10—C11	1.366 (3)
C3—H3	0.9300	C10—H10	0.9300
C4—C13	1.417 (3)	C11—C12	1.399 (3)
C4—C5	1.429 (3)	C11—H11	0.9300
C5—C6	1.347 (3)	C12—C13	1.445 (3)
C5—H5	0.9300	C14—H14	0.9300
C6—C7	1.426 (3)		
			
C1—N1—C13	117.6 (2)	C9—C8—C7	121.2 (3)
N1—C1—C2	125.7 (2)	C9—C8—H8	119.4
N1—C1—Cl1	115.3 (2)	C7—C8—H8	119.4
C2—C1—Cl1	119.0 (2)	C8—C9—C10	119.9 (3)
C3—C2—C1	116.6 (2)	C8—C9—H9	120.0
C3—C2—C14	118.8 (3)	C10—C9—H9	120.0
C1—C2—C14	124.5 (3)	C11—C10—C9	120.5 (3)
C2—C3—C4	120.8 (2)	C11—C10—H10	119.7
C2—C3—H3	119.6	C9—C10—H10	119.7
C4—C3—H3	119.6	C10—C11—C12	120.4 (3)
C3—C4—C13	117.7 (2)	C10—C11—H11	119.8
C3—C4—C5	123.3 (3)	C12—C11—H11	119.8
C13—C4—C5	119.0 (2)	C11—C12—C7	119.4 (2)
C6—C5—C4	120.6 (3)	C11—C12—C13	122.2 (2)
C6—C5—H5	119.7	C7—C12—C13	118.4 (2)
C4—C5—H5	119.7	N1—C13—C4	121.6 (2)
C5—C6—C7	122.1 (3)	N1—C13—C12	118.0 (2)
C5—C6—H6	119.0	C4—C13—C12	120.4 (2)
C7—C6—H6	119.0	O1—C14—C2	124.0 (3)
C8—C7—C12	118.5 (2)	O1—C14—H14	118.0
C8—C7—C6	121.9 (3)	C2—C14—H14	118.0
C12—C7—C6	119.6 (2)		
			
C13—N1—C1—C2	0.9 (4)	C9—C10—C11—C12	1.1 (4)
C13—N1—C1—Cl1	−179.99 (16)	C10—C11—C12—C7	−1.6 (4)
N1—C1—C2—C3	−0.6 (4)	C10—C11—C12—C13	177.6 (2)
Cl1—C1—C2—C3	−179.69 (17)	C8—C7—C12—C11	1.0 (3)
N1—C1—C2—C14	178.9 (2)	C6—C7—C12—C11	−179.3 (2)
Cl1—C1—C2—C14	−0.2 (3)	C8—C7—C12—C13	−178.2 (2)
C1—C2—C3—C4	−0.5 (3)	C6—C7—C12—C13	1.4 (3)
C14—C2—C3—C4	−180.0 (2)	C1—N1—C13—C4	−0.1 (3)
C2—C3—C4—C13	1.1 (3)	C1—N1—C13—C12	179.6 (2)
C2—C3—C4—C5	179.4 (2)	C3—C4—C13—N1	−0.8 (3)
C3—C4—C5—C6	−178.3 (2)	C5—C4—C13—N1	−179.2 (2)
C13—C4—C5—C6	0.0 (4)	C3—C4—C13—C12	179.5 (2)
C4—C5—C6—C7	−0.3 (4)	C5—C4—C13—C12	1.1 (3)
C5—C6—C7—C8	179.2 (2)	C11—C12—C13—N1	−0.7 (3)
C5—C6—C7—C12	−0.4 (4)	C7—C12—C13—N1	178.5 (2)
C12—C7—C8—C9	0.0 (4)	C11—C12—C13—C4	179.0 (2)
C6—C7—C8—C9	−179.6 (2)	C7—C12—C13—C4	−1.8 (3)
C7—C8—C9—C10	−0.6 (4)	C3—C2—C14—O1	10.7 (4)
C8—C9—C10—C11	0.0 (4)	C1—C2—C14—O1	−168.7 (3)
